# Feasibility of using negative ultrasonography results of axillary lymph nodes to predict sentinel lymph node metastasis in breast cancer patients

**DOI:** 10.1002/cam4.1606

**Published:** 2018-06-14

**Authors:** Xue Chen, Yingjian He, Jiwei Wang, Ling Huo, Zhaoqing Fan, Jinfeng Li, Yuntao Xie, Tianfeng Wang, Tao Ouyang

**Affiliations:** ^1^ Key Laboratory of Carcinogenesis and Translational Research (Ministry of Education) Breast Cancer Prevention & Treatment Center Peking University Cancer Hospital & Institute Beijing China

**Keywords:** axillary staging, breast cancer, receiver operating characteristic, sentinel lymph node biopsy, ultrasonography

## Abstract

Knowledge of the pathology of axillary lymph nodes (ALN) in breast cancer patients is critical for determining their treatment. Ultrasound is the best noninvasive evaluation for the ALN status. However, the correlation between negative ultrasound results and the sentinel lymph nodes (SLN) pathology remains unknown. To test the hypothesis that negative ultrasound results of ALN predict the negative pathology results of SLN in breast cancer patients, we assessed the association between ALN ultrasonography‐negative results and the SLN pathology in 3115 patients with breast cancer recruited between October 2010 and April 2016 from a single cancer center, prospective database. Of these patients who met the inclusion criteria, 2317 (74.4%) had no SLN pathological metastasis. In the univariate analysis, other 798 patient with positive SLN tended to be under age 40 and premenopausal, having large tumor sizes (>2 cm), higher histological grade of primary tumor, positive hormone receptors, and negative HER‐2 status (*P *<* *.05 for all). In the multivariate analysis, menstrual status, tumor size, ER status and histological types of primary tumor remained to be independent predictors for SLN pathological metastasis. The area under curve (AUC) was 0.658 (95% CI = 0.637‐0.679), *P* >* *.05. In conclusion, only a 74.4% consistency between ALN ultrasonography‐negative results and negative pathological SLN results, although menstrual status, tumor size, histologic subtypes of primary tumor and ER status were found to be statistically independent predictors of positive SLN among patients negative for ALN ultrasonography. Therefore, the present study suggests that negative ultrasound results of ALN do not adequately predict the negative pathology results of SLN in breast cancer patients.

## 1 INTRODUCTION

The pathological status of axillary lymph nodes (ALN) is critical in the treatment of breast cancer patients. Sentinel lymph node (SLN) biopsy is the standard of care for clinical management of breast cancer patients.^1^, ^2^, ^3^, ^4^ The SLN pathological result‐based treatment may prevent patients from undergoing unnecessary ALN dissection. Ultrasound is currently the best noninvasive method to evaluate ALN.^5^, ^6^, ^7^, ^8^ Due to improvements in ultrasonography equipment, advances in technology and refined diagnostic standards, the consistency between ALN ultrasonography‐negative results and negative SLN results has been improved. The SOUND (sentinel node vs observation after axillary UltraSouND) trial^9^ was established to compare SLN biopsy vs observation, when axillary ultra‐sound is negative in patients with small breast cancer sizes, who are candidates for breast conserving surgery. However, to our knowledge, few studies have evaluated the association between ALN ultrasonography‐negative results and pathological SLN results. Moreover, the influence of clinicopathological factors on SLN results in ALN ultrasonography‐negative patients has not been reported. Based on our single cancer center prospective database, we retrospectively analyzed the possible effects of age, menstrual status, primary tumor size, histologic subtypes of primary tumor, hormone receptor status, and HER‐2 status on SLN pathology results in breast cancer patients with ALN ultrasonography‐negative results, and evaluated the feasibility of predicting the SLN pathological results for ALN negative ultrasonography in these patients. We hypothesize that ultrasonography together with clinical indexes predict SLN metastasis for breast cancer patient with ALN ultrasonography‐negative results.

## 2 MATERIALS AND METHODS

### 2.1 Patients

Data were extracted from the prospective database of Peking University Cancer Hospital Breast Cancer Prevention and Treatment Center. All subjects included in the present study consisted of consecutively unselected patients treated between October 2010 and April 2016.

#### 2.1.1 Inclusion criteria

The primary tumor was diagnosed as an invasive breast cancer by core needle biopsy; ALN were ultrasonography‐negative; Patients underwent SLN biopsy in the same department; Patients were untreated before undergoing SLN biopsy.

#### 2.1.2 Exclusion criteria

The invasive breast cancer was pathologically diagnosed by surgical resection rather than by core needle biopsy; ALN were ultrasonography‐positive; Patients underwent treatment prior to SLN biopsy.

### 2.2 Ultrasonography

Axillary ultrasonography was performed by 3 sonographers who had at least 5 years of experience. Ultrasonography examinations were performed with an Acuson Antare unit (Siemens Medical Solutions, Mountain View, CA), a Mylab90 unit (Esaote, Genova, Italy) and a LOGIQ E9 unit (GE Healthcare, Milwaukee, WI) with transducers of 9 MHz or higher frequencies. ALN were divided into 2 groups: The ultrasonography negative axilla group including no ALN detected or a cortical thickness <3 mm. The definition of ALN‐positive ultrasonography results was consistent with the standards of the cancer center. ALN‐positive ultrasonography results were determined by one of the following 3 criteria.^10^ (1) Regular target annular lymph node with a peripheral cortical thickness ≥3 mm; (2) Eccentric target annular lymph node with a local cortical thickness ≥3 mm; and (3) Hypoechoic lymph node without a hilus structure of the lymph node.

### 2.3 SLN biopsy

The ^99m^Tc‐rituximab was used as the tracer for SLN biopsy. The tracer was injected into 2 of the following sites: the peritumoral breast parenchyma, subcutaneous and subareolar tissues. The injection dose was 18.5 MBq, injected in the morning of the surgery day, or 37 MBq on the day before surgery. SLN biopsy was performed under local anesthesia. SLN were identified by a handled gamma detection probe (Neoprobe, USA or Crystal, Germany). All radioactive nodes with a counting rate ≥10% of the hottest node were removed.

These data were used to correlate the results of ALN ultrasonography‐negative and SLN biopsy pathology and to be analyzed for possible influence of clinicopathological factors on SLN biopsy pathological results.

### 2.4 Statistical methods

In the univariate analysis, the patients’ age, menstrual status, primary tumor size, histological subtypes of primary tumor, hormone receptor status, and HER‐2 status were evaluated for the association with the SLN pathologic results. A Chi‐square test was used in the univariate analysis to evaluate the factors that influenced the SLN pathologic results, and a value of *P *<* *.05 was defined as statistically significant. In the multivariate analysis, factors that showed to be relevant to the SLN results in the univariate analysis were included in the multivariate logistic regression model. The receiver operating characteristic (ROC) curve employed to compare among variables for the ability to predict diagnostic results. Area under the ROC curve (AUC) was measured to test the prediction performance. AUC > 0.75 was defined as statistically significant.

## 3 RESULTS

Data were extracted from the prospective database, and all subjects included in the present study consisted of consecutively unselected patients treated between October 2010 and April 2016, resulting in a total of 5978 cases. According to the inclusion and exclusion criteria, 3115 patients with ALN ultrasonography‐negative results who underwent SLN biopsy were included in the comparison of the ALN ultrasonography‐negative results and SLN pathological results, including 2317 (74.4%) patients without SLN metastasis and 798 (25.6%) patients with SLN metastasis (Table 1).

**Table 1:** The clinicopathological characteristics of patients with ultrasonography‐negative ALN

Variables	Pathological SLN‐ n (%)	Pathological SLN+ n (%)	Total n (%)	*P* value
Age (years)				.036
≤40	324 (70.5)	136 (29.5)	460 (14.8)	
>40	1993 (75.1)	662 (24.9)	2655 (85.2)	
Menstrual state				.001
Premenopausal	1215 (71.8)	477 (28.2)	1692 (54.3)	
Postmenopausal	1102 (77.4)	321 (22.6)	1423 (45.7)	
Tumor size (cm)				.002
≤2	1242 (78.4)	342 (21.6)	1584 (50.9)	
>2	1075 (70.2)	456 (29.8)	1531 (49.1)	
Histology subtypes of primary tumor				<.001
IDC I	455 (83.0)	93 (17.0)	548 (17.6)	
IDC II & IDC III	1603 (71.1)	649 (28.8)	2252 (72.3)	
Others	259 (82.2)	56 (17.8)	315 (10.1)	
ER				<.001
Positive	1741 (71.4)	697 (28.6)	2438 (78.3)	
Negative	576 (85.0)	101 (15.0)	677 (21.7)	
PR				<.001
Positive	1670 (72.1)	646 (27.9)	2316 (74.3)	
Negative	647 (81.0)	152 (19.0)	799 (25.7)	
HER‐2				.008
Positive	588 (78.3)	163 (21.7)	751 (24.1)	
Negative	1729 (73.1)	635 (26.9)	2364 (75.9)	

ALN, axillary lymph nodes; ER, estrogen receptors; HER‐2, human epidermal growth factor receptor‐2; IDC, invasive ductal carcinoma; PR, progesterone receptor; SLN, sentinel lymph node.

**Figure 1:**
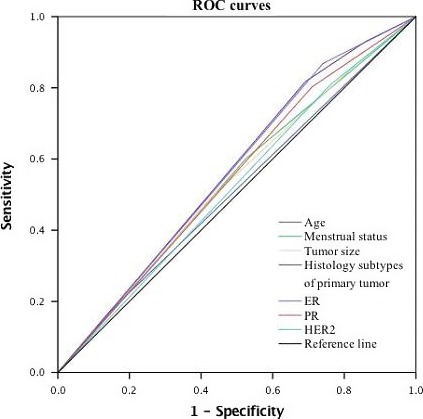
The ROC curves of the each factor

**Figure 2:**
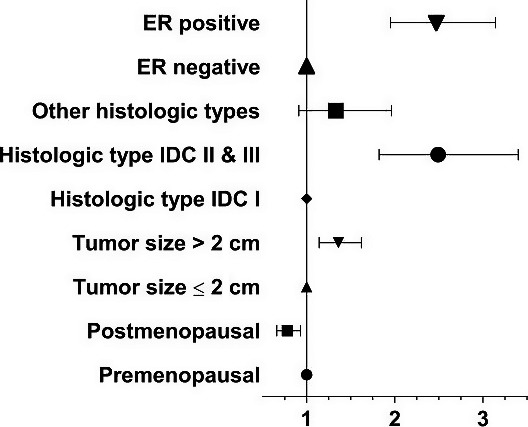
Forest plot showing adjusted ORs and 95% CIs for positive SLN in patients with ultrasonography negative axilla. Subgroups were defined by factors showing significant correlation with positive SLN. ER, estrogen receptors; IDC, invasive ductal carcinoma

Results of univariate analysis and multivariate analysis are listed in Tables 2, 3, 4 and Figures 1 and 2. In the univariate analysis for possible influence of clinicopathological factors on SLN biopsy results, patients over age 40 were less likely to have SLN metastasis, compared to patients under or equal to age 40 (*P *<* *.05, OR = 0.794, 95% CI = 0.638‐0.988). Postmenopausal patients were less prone to SLN metastasis than premenopausal patients (*P *<* *.05, OR = 0.743, 95% CI = 0.632‐0.874). Patients with tumors >2 cm were more likely to have SLN metastasis than those with tumors ≤2 cm (*P *<* *.05, OR = 1.296, 95% CI = 1.103‐1.522). The higher the tumor's histological grade was, the more likely the patients to have SLN metastasis (*P *<* *.05, OR = 1.958, 95% CI = 1.539‐2.492). Patients with positive ER were more likely to have SLN metastasis (*P *<* *.05, OR = 2.294, 95% CI = 1.823‐2.888). Patients with positive PR were more likely to have SLN metastasis (*P *<* *.05, OR = 1.649, 95% CI = 1.350‐2.012). Finally, HER‐2‐positive patients were less likely to have SLN metastasis than HER‐2‐negatice patients (*P *<* *.05, OR = 0.755, 95% CI = 0.612‐0.93).

**Table 2:** The univariate logistic regression model related to positive SLN in patients with ultrasonography‐negative ALN

Variables	OR	95% CI	*P* value
Age (years)			.039
≤40	1.00		
>40	0.79	0.64‐0.99	
Menstrual status			<.001
Premenopausal	1.00		
Postmenopausal	0.74	0.63‐0.87	
Tumor size (cm)			.002
≤2	1.00		
>2	1.30	1.10‐1.52	
Histology subtypes of primary tumor			<.001
IDC I	1.00		
IDC II & IDC III	1.96	1.54‐2.49	
Others	0.77	0.54‐1.10	
ER			<.001
Negative	1.00		
Positive	2.29	1.82‐2.89	
PR			<.001
Negative	1.00		
Positive	1.65	1.35‐2.01	
HER‐2			.008
Negative	1.00		
Positive	0.76	0.61‐0.93	

ALN, axillary lymph nodes; CI, confidence interval; ER, estrogen receptors; HER‐2, human epidermal growth factor receptor‐2; IDC, invasive ductal carcinoma; OR, odds ratio; PR, progesterone receptor; SLN, sentinel lymph node.

**Table 3:** The receiver operating characteristic (ROC) curves for the prediction of positive SLN with each clinical and pathological factor area

Variables	Area under the curve	95% CI
Age	0.513	0.489‐0.538
Menstrual status	0.538	0.514‐0.562
Tumor size	0.535	0.511‐0.559
Histology subtypes of primary tumor	0.565	0.542‐0.588
ER	0.564	0.541‐0.587
PR	0.546	0.523‐0.570
HER‐2	0.524	0.500‐0.548

AUC, area under curve; CI, confidence interval; ER, estrogen receptors; HER‐2, human epidermal growth factor receptor‐2; PR, progesterone receptor; SLN, sentinel lymph node.

**Table 4:** The multivariate logistic regression model related to positive SLN in patients with ultrasonography‐negative axilla

Variables	OR	95% CI	*P* value
Menstrual status			.006
Premenopausal	1.00		
Postmenopausal	0.78	0.66‐0.93	
Tumor size (cm)			.002
≤2	1.00		
>2	1.36	1.14‐1.62	
Histology subtypes of primary tumor			<.001
IDC I	1.00		
IDC II & IDC III	2.49	1.82‐3.40	
Others	1.33	0.91‐1.96	
ER			<.001
Negative	1.00		
Positive	2.47	1.95‐3.14	

CI, confidence interval; ER, estrogen receptors; IDC, invasive ductal carcinoma; OR, odds ratio; SLN, sentinel lymph node.

**Figure 3:**
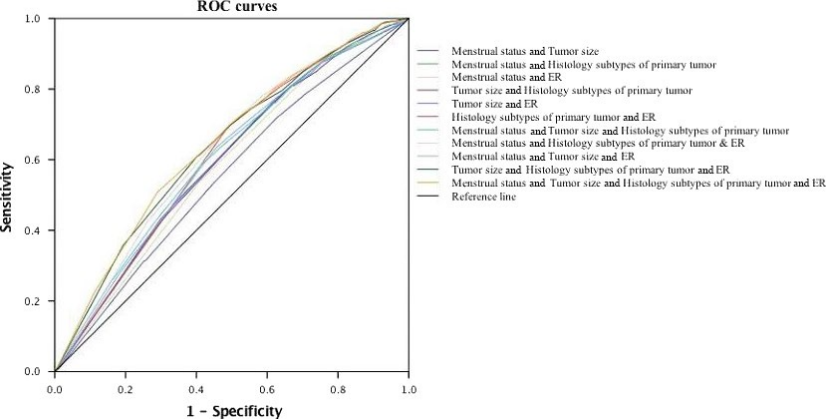
The ROC curves of the combination of each independent factors

Subsequently, multivariate analyses were carried out for those variables found to be statistically significant on univariate analyses. As a result, menstrual status, tumor size, ER status, and histologic subtypes of primary tumor remained to be independent predictors in the multivariate analyses (Table 4). Obviously, those significant predictors in univariate, but not in multivariate, analyses were correlated with those in the multivariate analyses. For example, age was highly correlated with menstrual status (*r* ^2^ = .146, *P *<* *.001), and both PR and HER‐2 were highly correlated with ER (*r* ^2^ = .444, *P *<* *.001 and *r* ^2^ = .062, *P *<* *.001, respectively). However, the AUC for the prediction model was 0.658 (95% CI = 0.637‐0.679), *P *>* *.05 (Table 5 and Figure 3).

**Table 5:** The ROC curves for the prediction of positive SLN by logistic models with the combination of each independent factors areas

Variables	AUC	95% CI
Menstrual status & Tumor size	0.555	0.532‐0.578
Menstrual status & Histology subtypes of primary tumor	0.600	0.578‐0.622
Menstrual status & ER	0.585	0.563‐0.607
Tumor size & Histology subtypes of primary tumor	0.600	0.578‐0.622
Tumor size & ER	0.597	0.575‐0.619
Histology subtypes of primary tumor & ER	0.614	0.592‐0.635
Menstrual status & Tumor size & Histology subtypes of primary tumor	0.612	0.590‐0.634
Menstrual status & Histology subtypes of primary tumor & ER	0.629	0.608‐0.651
Menstrual status & Tumor size & ER	0.606	0.583‐0.628
Tumor size & Histology subtypes of primary tumor & ER	0.635	0.613‐0.657
Menstrual status & Tumor size & Histology subtypes of primary tumor & ER	0.658	0.637‐0.679

AUC, area under curve; CI, confidence interval; ER, estrogen receptors; SLN, sentinel lymph node.

## 4 DISCUSSION

Axillary lymph nodes pathological status is important in the treatment strategy for breast cancer, and ALN evaluation includes several noninvasive and invasive methods, of which ultrasound has received much attention in modern breast cancer management, because it is noninvasive, highly sensitive to lymph nodes, indicates and guides further possible invasive examinations, and has a high pathological predictive value.^11^, ^12^ SLN biopsy is the gold standard for accurately evaluating ALN pathological status in breast cancer patients. The possibility of exempting routine SLN biopsy for patients with ALN ultrasonography‐negative results has become a focus of research.

In the present study of comparing patients of ALN‐negative ultrasound results with pathological SLN biopsy results, we found that among all subjects with ALN‐negative ultrasound results, 2317 cases without SLN pathological metastasis (74.4%) and 798 cases with SLN pathological metastasis (25.6%). This finding did not provide supporting evidence for our hypothesis, but rather indicated that if the negative SLN biopsy result was replaced by the ALN‐negative ultrasound results as the treatment basis, up to 25.6% of patients would be at risk of undertreatment due to underestimated SLN pathological metastasis. Thus, an ALN‐negative ultrasound result cannot replace SLN biopsy in accurately evaluating ALN status in breast cancer. Undertreatment may also affect the long‐term survival of patients with ALN‐negative ultrasound results. To our knowledge, this is the first report to compare data between ALN‐negative ultrasound results and negative pathological SLN biopsy results using a large sample in a Chinese population.

In addition to directly comparing the ALN‐negative ultrasound results and negative pathological SLN biopsy results, we also selected clinicopathological factors that are widely used to reflect malignancy in breast cancer. These clinicopathological factors included age, menstrual status, primary tumor size, pathological classification, hormone receptor status, and HER‐2 status. We attempted to determine whether combining these factors with ultrasound results would help select patients with ALN‐negative ultrasound results, who would possibly be exempted from SLN biopsy. Multivariate analyses showed that menstrual status, tumor size, ER status, and pathological types were found to be independently significant predictors. To our knowledge, no publication presented the relationship between menstrual status and SLN metastasis.

In the present study, premenopausal patients were more prone to SLN metastasis than postmenopausal patients, being 28.2% and 22.6%, respectively, (*P *<* *.05). Since menstrual status is closely correlated with patients’ age, both an age‐related time‐dependent exposure as well as hormonal effect could be a reasonable explanation. Kenney et al^13^ shows that the ALN metastasis drops in elder patients. Our data show that compared to patients over age 40, patients under age 40 were more likely to have SLN metastasis. Tumor size has long been believed to be an independent predictor of ALN metastasis.^14^, ^15^, ^16^, ^17^, ^18^ In the present study, patients with larger tumors (>2 cm) were more likely to have SLN metastasis than those with smaller tumors (≤2 cm). Furthermore, among the patients whose tumor was ≤2 cm (T1), the SLN metastasis rate is 0 (0 of 6) for T1a (≤0.5 cm) patients, 12.6% (28 of 222) for T1b (0.5‐1 cm) patients, and 23.17% (314 of 1356) for T1c (1.1‐2 cm) patients, which is in consistent with the results of other studies.^14^, ^15^, ^16^, ^17^, ^18^ In terms of pathological types, the present study showed that the higher the histological grade was, the greater SLN metastasis patients had. This is also in consistent with the results of other studies.^17^, ^18^, ^19^ As for the ER status, our study shows that patients with positive ER were more likely to have SLN metastasis, and patients with positive ER were more likely to be PR positive and HER‐2 positive as well. Tan et al^18^, ^20^ reported similar results. In the present study, we identified 4 statistically independent SLN status predictors with an AUC of 0.658, indicating that for patients with ultrasonography negative axilla, even combining these clinical and pathological factors cannot adequately predict the SLN status.

Since the prediction accuracy of SLN pathology for ALN status is as high as 97%, SLN biopsy avoids some of the unnecessary ALN dissection with huge trauma, but currently none of the minimally invasive procedures has reach this prediction accuracy alone or in combination. How to further reduce, and even avoid traumatic examination is the eternal pursuit in the strategy of breast cancer diagnosis and treatment. The present study had a goal to replace SLN biopsy by the noninvasive method, ultrasound, to evaluate ALN, but the results showed that the prediction accuracy of SLN pathology by ultrasound negative ALNs was only 74.4%, with an inaccuracy rate of 25.6%, in other words, which is unacceptable in clinical practice. However, we also observed that some clinical factors were predictive of SLN pathology, such as tumor size and ER status, among those patients with ultrasound negative ALNs. In future studies, we will collect additional detailed pathological data, such as sub‐classification of tumor size (T1a, T1b, and T1c), to increase and improve the prediction value of ultrasound ALN negative results for SLN pathology. Furthermore, we will consider incorporating some newly discovered predictive‐relevant molecules in oncology research.

Some of the characteristic of this present study are unique: (1) the time span for patient collection were relatively short so that the material, equipment, and technology were consistent; (2) the patients were treated by an experienced multidisciplinary breast cancer team, including ultrasound experts and SLN biopsy breast cancer surgeons with homogeneous specialized training; and (3) data were drawn from a large number of homogeneous patients with ALN‐negative ultrasound results.

However, the present study also has some limitations. Although the data were collected from the prospective database of a single cancer center, the nature of the present study is retrospective, selection bias is inherent. Further validation by larger, multicenter cohort as well as randomized controlled clinical trials is required.

In conclusion, the present study found a 74.4% consistency between ALN ultrasonography‐negative results and negative pathological SLN results. Although menstrual status, tumor size, histologic subtypes of primary tumor, and ER status were found to be statistically independent predictors of SLN pathological metastasis, the nonstatistically significant AUC indicates that the combination of ultrasonography, even with the clinic‐pathologic factors, did not generate an acceptable prediction accuracy.

## CONFLICT OF INTEREST

The authors declare that they have no conflict of interest. All authors have approved the final draft submitted.

## References

[1] Giuliano AE , Dale PS , Turner RR , et al. Improved axillary staging of breast cancer with sentinel lymphadenectomy. Ann Surg. 1995;222:394‐399. discussion 399‐401. 7677468 10.1097/00000658-199509000-00016 PMC1234825

[2] Veronesi U , Paganelli G , Viale G , et al. A randomized comparison of sentinel‐node biopsy with routine axillary dissection in breast cancer. N Engl J Med. 2003;349:546‐553. 12904519 10.1056/NEJMoa012782

[3] Veronesi U , Viale G , Paganelli G , et al. Sentinel lymph node biopsy in breast cancer: ten‐year results of a randomized controlled study. Ann Surg. 2010;251:595‐600. 20195151 10.1097/SLA.0b013e3181c0e92a

[4] Lyman GH , Temin S , Edge SB , et al. Sentinel lymph node biopsy for patients with early‐stage breast cancer: American Society of Clinical Oncology clinical practice guideline update. J Clin Oncol. 2014;32:1365‐1383. 24663048 10.1200/JCO.2013.54.1177

[5] Pamilo M , Soiva M , Lavast EM . Real‐time ultrasound, axillary mammography, and clinical examination in the detection of axillary lymph node metastases in breast cancer patients. J Ultrasound Med. 1989;8:115‐120. 2657088 10.7863/jum.1989.8.3.115

[6] Alvarez S , Anorbe E , Alcorta P , et al. Role of sonography in the diagnosis of axillary lymph node metastases in breast cancer: a systematic review. AJR Am J Roentgenol. 2006;186:1342‐1348. 16632729 10.2214/AJR.05.0936

[7] Feng Y , Huang R , He Y , et al. Efficacy of physical examination, ultrasound, and ultrasound combined with fine‐needle aspiration for axilla staging of primary breast cancer. Breast Cancer Res Treat. 2015;149:761‐765. 25667099 10.1007/s10549-015-3280-z

[8] Jackson RS , Mylander C , Rosman M , et al. Normal axillary ultrasound excludes heavy nodal disease burden in patients with breast cancer. Ann Surg Oncol. 2015;22:3289‐3295. 26224404 10.1245/s10434-015-4717-7

[9] Gentilini O , Veronesi U . Abandoning sentinel lymph node biopsy in early breast cancer? A new trial in progress at the European Institute of Oncology of Milan (SOUND: sentinel node vs Observation after axillary UltraSouND). Breast. 2012;21:678‐681. 22835916 10.1016/j.breast.2012.06.013

[10] Huo L , Yan K , Zhang H , et al. Morphology‐based criteria for ultrasonic assessment of axillary lymph node status in primary breast cancer. Zhonghua Yi Xue Za Zhi. 2012;92:672‐674. 22781293

[11] Deurloo EE , Tanis PJ , Gilhuijs KG , et al. Reduction in the number of sentinel lymph node procedures by preoperative ultrasonography of the axilla in breast cancer. Eur J Cancer. 2003;39:1068‐1073. 12736105 10.1016/s0959-8049(02)00748-7

[12] Choi YJ , Ko EY , Han BK , et al. High‐resolution ultrasonographic features of axillary lymph node metastasis in patients with breast cancer. Breast. 2009;18:119‐122. 19297159 10.1016/j.breast.2009.02.004

[13] Kenney RJ , Marszalek JM , McNally ME , et al. The effects of race and age on axillary lymph node involvement in breast cancer patients at a Midwestern safety‐net hospital. Am J Surg. 2008;196:64‐69. 18439985 10.1016/j.amjsurg.2007.09.035

[14] Barth A , Craig PH , Silverstein MJ . Predictors of axillary lymph node metastases in patients with T1 breast carcinoma. Cancer. 1997;79:1918‐1922. 9149018

[15] Carter CL , Allen C , Henson DE . Relation of tumor size, lymph node status, and survival in 24,740 breast cancer cases. Cancer. 1989;63:181‐187. 2910416 10.1002/1097-0142(19890101)63:1<181::aid-cncr2820630129>3.0.co;2-h

[16] Chadha M , Chabon AB , Friedmann P , et al. Predictors of axillary lymph node metastases in patients with T1 breast cancer. A multivariate analysis. Cancer. 1994;73:350‐353. 8293399 10.1002/1097-0142(19940115)73:2<350::aid-cncr2820730219>3.0.co;2-5

[17] Rivadeneira DE , Simmons RM , Christos PJ , et al. Predictive factors associated with axillary lymph node metastases in T1a and T1b breast carcinomas: analysis in more than 900 patients. J Am Coll Surg. 2000;191:1‐6. discussion 6‐8. 10898177 10.1016/s1072-7515(00)00310-0

[18] Yoshihara E , Smeets A , Laenen A , et al. Predictors of axillary lymph node metastases in early breast cancer and their applicability in clinical practice. Breast. 2013;22:357‐361. 23022046 10.1016/j.breast.2012.09.003

[19] Vandorpe T , Smeets A , Van Calster B , et al. Lobular and non‐lobular breast cancers differ regarding axillary lymph node metastasis: a cross‐sectional study on 4,292 consecutive patients. Breast Cancer Res Treat. 2011;128:429‐435. 21562708 10.1007/s10549-011-1565-4

[20] Tan LG , Tan YY , Heng D , et al. Predictors of axillary lymph node metastases in women with early breast cancer in Singapore. Singapore Med J. 2005;46:693‐697. 16308642

